# Gene expression and network-based analysis reveals a novel role for hsa-miR-9 and drug control over the p38 network in glioblastoma multiforme progression

**DOI:** 10.1186/gm293

**Published:** 2011-11-28

**Authors:** Rotem Ben-Hamo, Sol Efroni

**Affiliations:** 1 The Mina and Everard Goodman Faculty of Life Science, Bar Ilan University, 1 Keren-Hayesod St, Ramat-Gan, 52900, Israel

## Abstract

**Background:**

Glioblastoma multiforme (GBM) is the most common, aggressive and malignant primary tumor of the brain and is associated with one of the worst 5-year survival rates among all human cancers. Identification of molecular interactions that associate with disease progression may be key in finding novel treatments.

**Methods:**

Using five independent molecular and clinical datasets with a set of computational algorithms we were able to identify a gene-gene and gene-microRNA network that significantly stratifies patient prognosis. By combining gene expression microarray data with microRNA expression levels, copy number alterations, drug response and clinical data, combined with network knowledge, we were able to identify a single pathway at the core of glioblastoma.

**Results:**

This network, the p38 network, and an associated microRNA, hsa-miR-9, facilitate prognostic stratification. The microRNA hsa-miR-9 correlated with network behavior and presents binding affinities with network members in a manner that suggests control over network behavior. A similar control over network behavior is possible through a set of drugs. These drugs are part of the treatment regimen for a subpopulation of the patients that participated in the TCGA study and for which the study provides clinical information. Interestingly, the patients that were treated with these specific sets of drugs, all of which targeted against p38 network members, demonstrate highly significant stratification of prognosis.

**Conclusions:**

Combined, these results call for attention to p38 network targeted treatment and present the p38 network-hsa-miR-9 control mechanism as critical in GBM progression.

## Background

Glioblastoma multiforme (GBM) is the most common, aggressive and malignant primary tumor of the brain and is associated with one of the worst 5-year survival rates among all human cancers [1]. This tumor diffusely infiltrates the brain early in its course, making complete resection impossible. Advances in treatment for newly diagnosed GBM have led to current 5-year survival rates of 9.8%. Despite therapy, once GBM progresses, the outcome is uniformly fatal, with median overall survival historically less than 30 weeks [2].

Merging datasets from different studies bridges biases, leads to identification of robust survival factors [3] and eases concerns about the instability of mRNA data [4,5]. By combining different datasets, we can overcome biases such as batch effect and get closer to finding firm prognostic biomarkers. In the work presented here, we analyzed gene expression data from five independent publicly available glioblastoma datasets, four from the Gene Expression Omnibus (GEO) database [6] (from studies by Freije *et al*. [7], Murat *et al*. [8], Phillips *et al*. [9], and Lee *et al*. [10]) and one from The Cancer Genome Atlas (TCGA) [11].

Here, we take an approach that utilizes network graph structure and combine it with information on clinical outcome to identify curated networks that may serve as biomarkers for survival and/or to uncover molecular mechanisms that control disease course. To make use of network graph structure, we applied methods to merge expression data with network knowledge for the quantification of the network expression behavior [12]. Interaction and pathway information was obtained from The National Cancer Institute's Pathway Interaction Database [13]. We combined pathway metrics with clinical data to determine the association of network behavior with phenotype in the five independent datasets. The four GEO datasets consist of gene expression microarray and clinical outcome data (vital status), and the data provided by the TCGA (for 373 patients) comprise expression abundance through microarrays, copy number variation, and microRNA expression data.

Somatic copy number variations are extremely common in cancer, and detection and mapping of copy number abnormalities provides an approach for associating aberrations with disease phenotype and for localizing critical genes [14]. The role of microRNAs (miRNAs) in many human diseases is well established, and their ability to act as both therapeutic agents and disease prognostic biomarkers makes it important to understand this family of molecules [15]. By studying these molecular changes and their versatility, we can identify targets for sophisticated therapeutics approaches.

## Materials and methods

### Gene datasets

#### The Cancer Genome Atlas dataset

Data were obtained from TCGA. This dataset comprises molecular characterizations from 373 GBM patients. For each patient, the database provides copy number (level 2 data, 150 patients), microarray (level 2 data, 373 patients) and miRNA values (level 3 data, 373 patients). In addition, the following clinical data variables were recorded for each patient: age, gender, chemotherapy status and vital status. Copy number variation levels were obtained from the Human Genome CGH 244A microarray. This Agilent 244A platform shows the highest sensitivity among microarray oligonucleotide platforms, with a single element being sufficient to detect a single-copy alteration [16]. Comparative genomic hybridization arrays provide a means for quantitative measurement of DNA copy number aberrations and for mapping them directly on to genome sequences. A value of 0 (log 2 ratio) indicates a normal state, 1 indicates 2 copy gains and -1 indicates heterozygous deletion. A standard threshold for copy number alteration of > 0.3 for amplification, and < -0.3 for deletion was applied as previously described [17-19]. Gene expression was quantified using an Affymetrix HT Human Genome U133 Array Plate Set. The expression data were normalized by quintile normalization to produce robust multi-array average (RMA) expression values from the Affymetrix CEL files. Gene expression in all five datasets was analyzed on the RMA expression data. miRNA expression levels were quantified using the UNC Agilent miRNA 8 × 15K database, which contains expression values of 1, 510 miRNAs.

#### Gene Expression Omnibus datasets

##### Validation set 1

Validation set 1, from Freije *et al*. [7], is composed of gene expression and clinical information from 74 GBM patients (GEO accession [GSE4412]). All patients were at grade III and IV, and ages varied from 18 to 82 years. The study included 46 females and 28 males. Gene expression was quantified using the Affymetrix Human Genome U133A Array.

##### Validation set 2

Validation set 2, from Lee *et al*. [10], is composed of gene expression and clinical information from 191 GBM patients (GEO accession [GSE13041]). Gene expression was quantified using the Affymetrix Human Genome U133A Array.

##### Validation set 3

Validation set 3, from Murat *et al*. [8], is composed of gene expression and clinical information from 80 GBM patients (GEO accession [GSE7696]). Gene expression was quantified using the Affymetrix Human Genome U133 Plus 2 Array.

##### Validation set 4

Validation set 4, from Phillips *et al*. [9] and Costa *et al*. [20], is composed of gene expression and clinical information from 77 GBM patients (GEO accession [GSE4271]). Gene expression was quantified using the Affymetrix Human Genome U133A Array.

#### Pathway network interactions dataset

Network information was obtained from the National Cancer Institute's Pathway Interaction Database [12].

### Gene expression analysis

Pathway consistency and pathway activity metrics were calculated according to [12,21]. These measures treat the pathway as a network of interactions and give the network a score based on the expression levels of each of the genes in the interaction and on the quality of the interaction. The analysis takes into consideration the specific type of interaction (such as inhibition or promotion).

The activity metric is a measure of the likelihood that the interaction occurs in the pathway. When taking a pathway with two genes as input and one gene as output, the algorithm calculates their probability of being in an 'up' state (by taking into account the expression levels of those genes in all the samples). The activity of this pathway is the probability that this interaction is 'active', meaning the product of the probabilities that the two genes are in the 'up' state. The consistency metric is a measure comparing the expected versus actual expression of the interaction components, obtained by calculating the probabilities (i) of an active interaction, (ii) that the output gene is in an 'up' state, and (iii) of the complementary event.

### Survival analysis

Kaplan-Meier survival analysis was done on all pathway measurements for all five datasets (by SPSS software, SPSS Inc. Chicago, IL, USA), using clinical data (vital status) to determine a pathway's survival stratification power. Log-rank tests were used to test the difference between survival groups; in all analyses a *P*-value < 5.0 e^-2 ^was accepted as significant. This analysis was done in order to identify pathways that could stratify prognosis in all five datasets.

All values (pathway activity and consistency) were clustered using K-means clustering to stratify the patients into two distinct groups according to their pathways values. Kaplan-Meier survival analysis was performed using the groups that emerged from this K-means clustering and using the clinical outcome data (vital status). Pathways that showed significant Kaplan-Meier *P*-values (< 0.05) were then tagged as successful stratification metrics for prognosis. All the results were then compared between the five datasets to identify overlapping pathways.

Kaplan-Meier survival analysis was also performed on all combinations of three drug sets; overall there were 249, 984 different combinations (constructed out of 64 drugs). In every iteration, the algorithm gathered all the patients that received one of the three drugs in question and calculated the Kaplan-Meier survival *P*-value for the generated group. Combinations that are associated with less than 20% of the patients were removed from the analysis as being insufficient. All combinations with significant *P*-values are shown in Additional file 1.

### False discovery rate for the p38 pathway

To determine whether the behavior of the p38 pathway across the five independent datasets was greater than expected by chance, the survival times in each of the five datasets were scrambled and randomly assigned to each patient. We performed clustering using k-means and calculated Kaplan-Meier log-rank *P*-values (as described earlier). We performed this renormalization five times to achieve a substantial sample (the results are shown in Additional file 2).

Not a single pathway consistently stratified prognosis in all five iterations in the five datasets. This demonstrates a 0% chance to identify a common pathway in all five different datasets and a 100% chance to find 0 pathways. Thus, the identification of the p38 pathway is unlikely to occur by chance.

## Results

**Figure 1:**
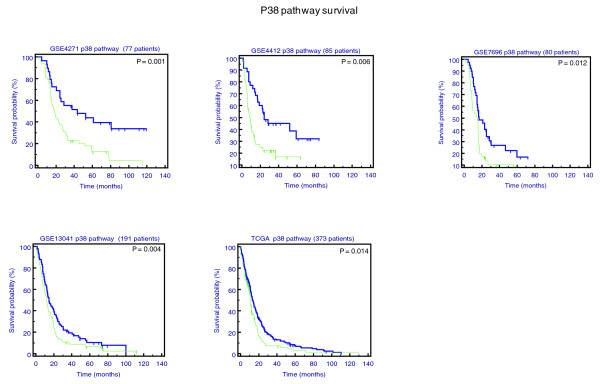
**Correlation between p38 pathway activity and patient survival**. Kaplan-Meier curves generated according to values for the p38 pathway across all five datasets. Across the five panels, group 1 (blue line), which is associated with better prognosis, shows lower pathway activity values and group 2 (green line) shows higher pathway activity values. The association of pathway metric levels with prognosis is highly robust in this case, as it shows low *P*-values and consistent behavior across datasets.

We found that the p38 network significantly and robustly stratifies prognosis in all five datasets (Figure 1). Importantly, none of the genes in this pathway, by themselves, show any statistical power in survival analysis; that is, the gene components of the network, when taken separately and out of the network context of the other genes in the pathway, fail to provide biomedical meaning. In addition, groups stratified by the network analyses we present here do not show any correlation with any clinical features. This furthers strengthens the hypothesis that this network is intrinsically involved in the development of GBM.

### Pathway analysis

To utilize knowledge of network graph structure, we applied methods for merging expression data with network information [12]. These methods quantify expression behavior in specific subnetworks (such subnetworks can be specific pathways or any other defined subnetwork) and produce two metrics: pathway activity and pathway consistency. In brief, a pathway's activity is a measure of how likely the interactions within a network are to be active in the specific sample at hand. A sample's pathway consistency measure is a measure of the compatibility between gene expression abundance in that sample and molecular description as detailed in the pathway's graph. Further details are given in [12].

To apply this network-based methodology, we used gene expression data from all five datasets described above and made use of these expression levels to deduce pathway metrics. Each sample was thus re-represented using its network metrics. This representation assigns 579 network metric scores (a score for each pathway in the database) to each sample in every dataset. Network information was obtained from The National Cancer Institute's Pathway Interaction Database [13]. We then iterated across the set of samples, using the network scores, to assign Kaplan-Meier *P*-values for each of the pathways. This procedure allows us to rank each of the pathways according to their ability to stratify patients into prognosis groups. We then combined all results in the five datasets in order to find overlapping pathways.

Following this procedure, we were able to identify one robust pathway that stratified prognosis across all five different sources of datasets; the p38 pathway demonstrated consistent behavior across all datasets. Further, this p38 network demonstrated highly significant biomarker abilities by stratifying prognosis. Figure 1 illustrates Kaplan-Meier survival across the dataset sources.

**Figure 2:**
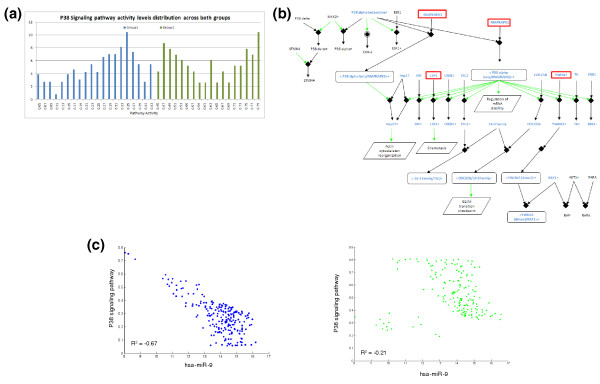
**hsa-miR-9 regulation of the p38 pathway**. **(a) **Distribution of p38 signaling pathway activity levels across groups. Group 1 (blue, higher survival rates) has low pathway activity, and group 2 (green, lower survival rates) has higher activity levels. This figure demonstrates the large range in the activity levels between groups, and the distinct difference between them. **(b) **The p38 signaling network. Genes highlighted in blue are in the p38 signaling pathway, and the genes in red boxes are those found by PITA to be possibly targeted by hsa-miR-9. **(c) **Correlation between hsa-miR-9 and p38 pathway levels. Group 1 (blue dots) has a significant, strong negative correlation between miRNA expression levels and pathway activity while group 2 (green dots) has a lower correlation value. The groups presented here are based on the p38 survival groups.

This pathway, when highly activated, associates with poor prognosis. This is in agreement with previous works that found that this pathway induces migration of glioblastoma cells when it is highly activated [22]. The pathway activity score is a value between 0 and 1 (see above), and the p38 network, in the context of the GBM samples studied, demonstrates highly variable values, from 0.05 up to 0.79, which result from variability in expression behavior of the genes in this network. Despite the range of values, however, the network metric remains robust enough to separate patients into two distinct groups. Figure 2 illustrates the difference in the p38 network metric between the two identified clinical groups.

The false discovery rate calculated using the intersection of all five datasets (as described in Materials and methods) was 0%, which means that identifying a single robust pathway (out of 579 different pathways) that significantly stratifies prognosis in five independent datasets could not occur by chance alone.

### Copy number variation analysis

To further study the molecular characteristics of this pathway, we made use of the intensive molecular features available through TCGA, which provides genetic information for each tumor sample. We analyzed copy number profiles of the p38 pathway genes. Using a Mann-Whitney U test, a non-parametric test that assess whether two independent samples have equally large values, we examined copy number aberrations in tumors and matched normal samples to see if copy number variations in these, for each specific gene, are independent samples from identical continuous distributions with equal medians, rather than the alternative that they do not have equal medians.

Probe sets with an inferred log2 ratio of > 0.3 or < -0.3 were classified as gains and losses, respectively. This analysis revealed that 11 out of the 13 genes in this pathway are highly targeted for copy number changes (*P*-value < 0.05; Table 1). Five of the genes were significantly amplified and six were deleted compared to the normal samples. These results reveal that the pathway is highly targeted by genomic variation, which may account, in part, for the demonstrated robust connection with disease outcome.

**Table 1:** Copy number variation profile of the p38 pathway

Gene symbol	Tumor	Normal
Amplified genes		
*HSP27*	21%	2%
*CREB1*	27%	16%
*TCF3*	14%	2%
*ER81*	45%	6%
*CDC25B*	36%	20%
Deleted genes		
*MAPKAPK3*	20%	11%
*LSP1*	31%	25%
*TH*	37%	14%
*YWHAZ*	63%	27%
*ALOX5*	68%	7%
*RAF1*	13%	9%

Of the 13 genes in the p38 pathway, 11 show significant change (according to Mann-Whitney U test) in copy number, amplification or deletion, between the tumor and its matched normal sample across all patients.

### miRNA analysis

miRNAs are known to be involved in the regulation of transcription in a complex manner [23]. As TCGA provides quantification of miRNA abundance for many samples, we were able to combine quantified network metrics with abundance levels of 1, 510 miRNAs to identify miRNAs that show significant correlation with network behavior and can thus be further studied as network control mechanism regulators.

Previous studies have shown the control function of miRNAs over pathways [24-27]. miRNAs hold the ability to simultaneously target and regulate many cellular pathways, the most noticeable of which control developmental and oncogenic processes. Notably, miRNA processing defects also enhance tumorigenesis. Interestingly, we were able to find significant negative correlation (*P*-value < 0.0001) between the p38 network and the miRNA hsa-miR-9. Further, gene sequences revealed that 4 out of the 13 genes in the pathway have a possible binding site for hsa-miR-9 (this analysis was performed using PITA [28], a prediction algorithm for potential miRNA targets). Possible binding between hsa-miR-9 and genes within the p38 pathway strengthen the hypothesis that miR-9 may indeed be a key regulator over pathway behavior and may serve as a potential therapeutic target for GBM patients.

### Drug target analysis

Over the past 25 years and despite vigorous basic and clinical studies, the median survival of patients with GBM remains low. The dataset from TCGA contains a significant body of clinical data that includes the type of treatment each patient received.

Different from the single gene perspective, pathways, constructed out of multiple genes that interact with one another in a combinatorial manner, contribute to phenotype in a more complex manner. The key argument here is that the function of a pathway is entirely defined by molecular interactions that take place between its components. Therefore, pathway targeting can be performed in different ways; it could be directed towards different key genes and still lead to similar phenotypes. Specifically, the regulation of the p38 pathway by hsa-miR-9 may be mimicked by different pharmaceutical components already in use.

**Figure 3:**
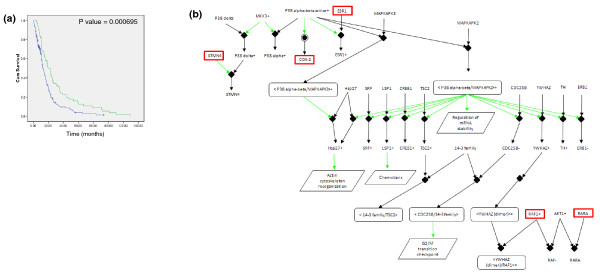
**Drug control over the p38 pathway**. **(a) **Kaplan-Meier curves generated according to the treatment profiles of the patients. Group 2 (green line), which is associated with better prognosis, consists of 63 patients who received one of the six drugs that target genes in the p38/MAPKAP pathway. Group 1 (blue line), which is associated with poorer prognosis, consists of 169 patients, none of whom received any of the six drugs. **(b) **The p38 signaling network. Genes highlighted with red boxes are those targeted by the six drugs (also see Table 2).

To investigate if drug regimen does control this pathway's behavior, we identified drugs that target genes in the p38 pathway and may lead to a phenotype similar to the one induced by hsa-miR-9 activity. Data on drugs administered to GBM patients in TCGA cover 64 unique drugs. Using DrugBank [29], a bioinformatics/chemoinformatics resource that combines detailed drug data with comprehensive drug target information, we were able to filter these drug targets into two groups: drugs that target genes that are part of the p38/mitogen-activated protein kinase-activated protein kinase (MAPKAP) pathway; and drugs whose targets are not included in the p38/MAPKAP pathway (Additional file 3). Using this simple classification, we tagged six drugs that target genes in the p38/MAPKAP pathway. Table 2 lists these drugs and their associated target genes, together with the pathway of which the genes are members. To learn about the clinical relevance of this pharmaceutical intervention, we divided patients into two groups: group 1, whose members received treatment with one of the six drugs that target the network; and group 2, whose members did not receive treatment with drugs that target the network. Using these groups as the basis for a Kaplan-Meier analysis, we see a highly significant (*P*-value < 0.0001) prognosis stratification (Figure 3). In clinical terms, this means that patients who were administered one of the six drugs that target genes in the p38/MAPKAP pathway had significantly higher survival rates than patients who were not: patients in group 1 had an average survival time of 896 days with a median survival of 691 days, while patients in group 2 had an average survival time of 433 days and a median survival time of only 310 days.

**Table 2:** Glioblastoma drug targets

Drug name	Target	Pathway
Accutane	RARA	MAPK inactivation of SMRT co-repressor
CCNU	STMN4	Signaling mediated by p38-gamma and p38-delta pathway
Celebrex	COX2	Signaling mediated by p38-alpha and p38-beta pathway
Cis-retinoic acid	RARA	MAPK inactivation of SMRT co-repressor
Sorafenib	RAF1	p38 signaling mediated by MAPKAP kinases
Tamoxifen	ESR1	Signaling mediated by p38-alpha and p38-beta pathway

The table lists 6 of the 69 drugs in TCGA clinical dataset that target genes in the p38/MAPKAP pathway. MAPK, mitogen-activated protein kinase.

GBM patients usually receive a broad spectrum of drugs from chemotherapy to hormonal therapy. All of the patients in this study received several drug regimens with no pattern of combination; the only common denominator were the six drugs described above. To validate that the combination of drugs we found is indeed the most significant one, we performed survival analysis on all combinations of sets of three drugs, and a Kaplan-Meier test was performed across all 249, 948 possible combinations (significant *P*-values are given in Additional file 1). Interestingly, after removing all combinations that are associated with less than 20% of the patients, we obtained 577 combinations that significantly stratified prognosis. Most importantly, however, the combination of drugs that targets the p38 pathway was more significant than that found by the exhaustive search.

The significant difference in survival times and the high significance in prognosis stratification between treatments that target the p38 pathway and those that do not strengthen the hypothesis that the p38 network is critical in the progression of GMB and perhaps in its development. Specific care should be given in view of these results with regard to treatments used in future clinical studies.

## Discussion

Auffray, Chen and Hood recently suggested that 'Systems approaches will transform the way drugs are developed through academy-industry partnerships that will target multiple components of networks and pathways perturbed in diseases' [30]. The work described here is an effort to take up this challenge.

Merging datasets from different studies leads to identification of robust survival factors, and applying tests that predict clinical outcome for patients based on RNA abundance in tumors is likely to increasingly affect patient management, heralding a new era of personalized medicine [31]. The consortium that is behind TCGA is the first to provide the community with population-sized, high-throughput molecular classification of diseases. This unique data resource, large portions of which are publicly available, offers a never before seen view of a disease's landscape.

Cancer is a disease of genomic alterations; changes in DNA sequence and genomic variations in copy number together provide a scaffold for the development and progression of malignancies. GBM is no different, although the clinical value of most GBM-associated molecular aberrations in terms of their significance for diagnosis and prognosis or as predictive molecular markers has remained unclear [32]. A better understanding of the molecular characteristics and biology of GBM may help improve treatment and identification of cellular factors that drive prognosis, and may also provide clues to novel treatments.

The genome-wide quantification of gene expression levels allows us to make the transition from single-gene-based research to molecular network-based analysis. Genome-wide details of genomic variation facilitate association of gained network knowledge with copy number variation, and abundance levels of miRNAs further provide a means to observe connections between such small RNAs, control networks and genomic variation. Finally, proper documentation of clinical data enables the rendition of network and molecular findings into translational medicine.

The results we present here demonstrate that these molecular networks, when scrutinized using the proper perspectives, enable associations between clinical and network modification data by stratifying patients' prognosis according to the molecular characterization of their tumors. Specifically, by first identifying the p38 transcription network as critical in disease outcome, by following this identification to uncover a possible regulatory mechanism involving the miRNA hsa-mir-9, and to finally match drug response to this network behavior, we reveal the clinical relevance of the p38-miR9 network and call for continued clinical scrutiny of it.

As we see here, patients in which hsa-miR-9 controls the p38 network in an efficient manner have better prognosis, and patients in which this hsa-miR-9 control fails have poorer prognosis. Interestingly, the same phenomenon is evident when considering drug control over the network; patients that receive drugs that target and inactivate the network have better prognosis, perhaps in a similar manner to that confered by hsa-miR-9. To support pathway behavior and to demonstrate its robustness as a clinical biomarker, we demonstrate that the same network behavior associates patients with outcome, regardless of specific batches of experimental procedures.

Through better understanding of the pathway mechanisms and the interactions that undergo changes, we may find targets for new treatments. The fact that the pathway we identified did not correlate with gender, age or chemotherapy status and was found in all five datasets strengthens the hypothesis that this pathway is a core mechanism of GBM.

## Conclusions

Integrating multidimensional, disease-specific, high-throughput data in the context of RNA control networks and their relevant drug responses provides an initial response to the biomedical community's appeal to identify pathways critically involved in disease outcome (for example, [33]). The transition from gene-disease to network-disease thinking is in need of further impetus. A careful clinical follow-up on findings such as those presented here, combined with careful molecular investigation of gene control mechanisms, such as the relations suggested here, could lead to the discovery of novel biomarkers and novel therapies. Further, an important point raised by Emmert-Streib and Glazko [34] is that the network as a 'conceptual framework' is, in and of itself, a way of thinking that may become an important systems biology paradigm in medical thinking. Network target identification, together with novel means to construct drug-target networks [35], are to advance rational drug discovery.

## Abbreviations

GBM: glioblastoma multiforme; GEO: Gene Expression Omnibus; MAPKAP: mitogen-activated protein kinase-activated protein kinase; miRNA: microRNA; TCGA: The Cancer Genome Atlas.

## Competing interests

The authors declare that they have no competing interests.

## Authors' contributions

RBH and SE designed the study, analyzed data and wrote the paper. All authors read and approved the final manuscript.

## Supplementary Material

Additional file 1 **Table S1 - the most significant three-drug combinations with corresponding *P*-values**. The *P*-value represents significance with regard to stratification of prognosis according to Kaplan-Meier survival analysis. Click here for file

Additional file 2 **Table S2 - all drugs with their corresponding gene targets**. This table also indicates whether there is a connection or not between a drug and the p38 pathway, and provides the number of patients who received each drug. Click here for file

Additional file 3 **Figure S1 - heat maps describing the false discovery rate analysis**. The five heat maps illustrate the five iterations that were performed. Every row represents a different pathway (overall 579 pathways), and columns represent the five datasets tested. A black line indicates a significant *P*-value (< 0.05) in Kaplan-Meier survival analysis. The heat map at the bottom shows the actual analysis that was performed with the p38 pathway as significant for survival in all five sets. Click here for file
