# Supplementary material for: Gene expression and network-based analysis reveals a novel role for hsa-miR-9 and drug control over the p38 network in glioblastoma multiforme progression
Source: Genome Med. 2011 Nov 28;3(11):77. doi: 10.1186/gm293 (PMC3308032; doi:10.1186/gm293)
Supplement: Additional file 1 — Table S1 - the most significant three-drug combinations with corresponding P-values. The P-value represents significance with regard to stratification of prognosis according to Kaplan-Meier survival analysis. [file gm293-S1.DOC]

| Drug A | Drug B | Drug C | Kaplan-Meier P-value |
| --- | --- | --- | --- |
| Gliadel Wafer | EMD-121974 | Dexamethasone | 0.001433 |
| Gliadel Wafer | SAHA | Dexamethasone | 0.002135 |
| Gliadel Wafer | Marimastat | Dexamethasone | 0.002321 |
| BCNU | CCNU | Xeloda | 0.002452 |
| Gliadel Wafer | IL-13 | Dexamethasone | 0.002455 |
| Gliadel Wafer | Daclizumab | Dexamethasone | 0.002473 |
| Gliadel Wafer | Dexamethasone | Angiocept | 0.002473 |
| Gliadel Wafer | CRA | Dexamethasone | 0.00251 |
| Gliadel Wafer | Dexamethasone | Panzem | 0.002529 |
| Everolimus | Gliadel Wafer | Dexamethasone | 0.002782 |
| Gliadel Wafer | AMG 102 | Dexamethasone | 0.002806 |
| BCNU | CCNU | Procarbazine | 0.002919 |
| BCNU | Gleevec | CCNU | 0.002985 |
| Celebrex | BCNU | CCNU | 0.003096 |
| Oxaliplatin | Gliadel Wafer | Dexamethasone | 0.003144 |
| Rapamcyin | Gliadel Wafer | Dexamethasone | 0.003144 |
| Gliadel Wafer | Chloroquine | Dexamethasone | 0.003144 |
| Gliadel Wafer | 6-Mercaptopurine | Dexamethasone | 0.003144 |
| Gliadel Wafer | Dexamethasone | Prednisone | 0.003144 |
| Gliadel Wafer | Dexamethasone | Bortezomib | 0.003144 |
| Gliadel Wafer | Dexamethasone | Streptozocin | 0.003144 |
| Gliadel Wafer | Dexamethasone | CI980 | 0.003144 |
| Gliadel Wafer | Dexamethasone | Fotemustine | 0.003144 |
| Gliadel Wafer | Dexamethasone | AE788 | 0.003144 |
| Gliadel Wafer | Dexamethasone | Motexafin gadolinium | 0.003144 |
| Gliadel Wafer | Dexamethasone | Arsenic Trioxide | 0.003144 |
| Gliadel Wafer | Dexamethasone | Carboxyl amino triazole | 0.003144 |
| Gliadel Wafer | Dexamethasone | Topotecan | 0.003316 |
| Gliadel Wafer | Dexamethasone | HSPPC-96 Vaccine | 0.003351 |
| BCNU | CCNU | Antineoplastons | 0.003626 |
| BCNU | Accutane | CCNU | 0.003763 |
| Iressa | Gliadel Wafer | Dexamethasone | 0.003834 |
| Gliadel Wafer | Dexamethasone | BSI-201 | 0.003999 |
| Gliadel Wafer | Dexamethasone | Recentin | 0.004153 |
| Gliadel Wafer | Dexamethasone | Suramin | 0.004194 |
| Gliadel Wafer | Dexamethasone | MGI-114 | 0.004194 |
| Gliadel Wafer | Dexamethasone | Enzastaurin | 0.004297 |
| BCNU | CCNU | dcVax | 0.004355 |
| BCNU | Hydroxurea | CCNU | 0.004504 |
| BCNU | CCNU | Thalidomide | 0.004568 |
| BCNU | Cis Retinoic Acid | CCNU | 0.004571 |
| Gliadel Wafer | O6BG | Dexamethasone | 0.004575 |
| Gliadel Wafer | Dexamethasone | Dendriticcell Vaccine | 0.004809 |
| Sarasar | BCNU | CCNU | 0.00485 |
| Rindopepimut | Gliadel Wafer | Dexamethasone | 0.00505 |
| Gliadel Wafer | 6-Thiguanine | Dexamethasone | 0.005143 |
| BCNU | Prednisone | CCNU | 0.005588 |
| BCNU | Sorafenib | CCNU | 0.005588 |
| BCNU | CCNU | Arsenic Trioxide | 0.005588 |
| BCNU | CCNU | Fotemustine | 0.005762 |
| BCNU | CCNU | Dendriticcell Vaccine | 0.005798 |
| Carboplatin | BCNU | CCNU | 0.005853 |
| CCNU | CPT11 | Xeloda | 0.005985 |
| Rindopepimut | BCNU | CCNU | 0.006202 |
| EMD-121974 | Dexamethasone | Thalidomide | 0.006301 |
| BCNU | BSI-201 | CCNU | 0.006424 |
| Cisplatin | Gliadel Wafer | Dexamethasone | 0.006807 |
| Sarasar | Gliadel Wafer | Dexamethasone | 0.006846 |
| Gleevec | CCNU | CPT11 | 0.007044 |
| Gliadel Wafer | Interferon Alpha | Dexamethasone | 0.007183 |
| Gliadel Wafer | Dexamethasone | Sorafenib | 0.007183 |
| Iressa | BCNU | CCNU | 0.007237 |
| Rapamycin | BCNU | CCNU | 0.007281 |
| BCNU | CCNU | AE788 | 0.007285 |
| Rapamcyin | BCNU | CCNU | 0.007306 |
| BCNU | Chloroquine | CCNU | 0.007306 |
| BCNU | 6-Mercaptopurine | CCNU | 0.007306 |
| BCNU | 6-Thiguanine | CCNU | 0.007306 |
| BCNU | Interferon Alpha | CCNU | 0.007306 |
| BCNU | O6BG | CCNU | 0.007306 |
| BCNU | Marimastat | CCNU | 0.007306 |
| BCNU | AP23573 | CCNU | 0.007306 |
| BCNU | Suramin | CCNU | 0.007306 |
| BCNU | Streptozocin | CCNU | 0.007306 |
| BCNU | CCNU | Topotecan | 0.007306 |
| BCNU | CCNU | Vincristine | 0.007306 |
| BCNU | CCNU | CI980 | 0.007306 |
| BCNU | CCNU | Recentin | 0.007306 |
| BCNU | CCNU | MGI-114 | 0.007306 |
| BCNU | CCNU | Motexafin gadolinium | 0.007306 |
| BCNU | CCNU | Carboxyl amino triazole | 0.007306 |
| Oxaliplatin | BCNU | CCNU | 0.007565 |
| BCNU | Bortezomib | CCNU | 0.007565 |
| 6-Thiguanine | Dexamethasone | Cis Retinoic Acid | 0.007609 |
| BCNU | Enzastaurin | CCNU | 0.007741 |
| Gliadel Wafer | Dexamethasone | dcVax | 0.007787 |
| Gleevec | CCNU | Procarbazine | 0.007861 |
| BCNU | CCNU | CPT11 | 0.008168 |
| CCNU | CPT11 | Procarbazine | 0.008306 |
| BCNU | CCNU | HSPPC-96 Vaccine | 0.008312 |
| Gliadel Wafer | Dexamethasone | Cis Retinoic Acid | 0.008368 |
| Cisplatin | BCNU | CCNU | 0.008447 |
| Dexamethasone | Thalidomide | CI980 | 0.008501 |
| BCNU | AMG 102 | CCNU | 0.008541 |
| CCNU | CPT11 | Antineoplastons | 0.008558 |
| Everolimus | BCNU | CCNU | 0.008578 |
| SAHA | Dexamethasone | Thalidomide | 0.008853 |
| Accutane | CCNU | CPT11 | 0.008894 |
| BCNU | Panzem | CCNU | 0.009006 |
| BCNU | CCNU | Cyclophosphamide | 0.009012 |
| BCNU | CRA | CCNU | 0.009041 |
| BCNU | Daclizumab | CCNU | 0.009109 |
| BCNU | CCNU | Angiocept | 0.009109 |
| BCNU | IL-13 | CCNU | 0.009142 |
| Marimastat | Dexamethasone | Thalidomide | 0.009457 |
| BCNU | SAHA | CCNU | 0.009801 |
| IL-13 | Dexamethasone | Thalidomide | 0.009884 |
| Daclizumab | Dexamethasone | Thalidomide | 0.00994 |
| Dexamethasone | Thalidomide | Angiocept | 0.00994 |
| CRA | Dexamethasone | Thalidomide | 0.010057 |
| Sarasar | Dexamethasone | Cis Retinoic Acid | 0.010079 |
| Dexamethasone | Panzem | Thalidomide | 0.010117 |
| Gliadel Wafer | AP23573 | Dexamethasone | 0.010138 |
| BCNU | CCNU | 81C6 | 0.010205 |
| CCNU | CPT11 | dcVax | 0.010222 |
| Hydroxurea | CCNU | Xeloda | 0.010429 |
| Everolimus | Dexamethasone | Thalidomide | 0.010907 |
| AMG 102 | Dexamethasone | Thalidomide | 0.01098 |
| Gliadel Wafer | Dexamethasone | Accutane | 0.011243 |
| CCNU | Thalidomide | Xeloda | 0.011304 |
| Hydroxurea | CCNU | Procarbazine | 0.011618 |
| Celebrex | EMD-121974 | Dexamethasone | 0.011813 |
| Cis Retinoic Acid | CCNU | CPT11 | 0.01197 |
| Hydroxurea | CCNU | CPT11 | 0.012156 |
| Oxaliplatin | Dexamethasone | Thalidomide | 0.012303 |
| Rapamcyin | Dexamethasone | Thalidomide | 0.012303 |
| 6-Mercaptopurine | Dexamethasone | Thalidomide | 0.012303 |
| Interferon Alpha | Dexamethasone | Thalidomide | 0.012303 |
| Dexamethasone | Prednisone | Thalidomide | 0.012303 |
| Dexamethasone | Bortezomib | Thalidomide | 0.012303 |
| Dexamethasone | Sorafenib | Thalidomide | 0.012303 |
| Dexamethasone | Streptozocin | Thalidomide | 0.012303 |
| Dexamethasone | Thalidomide | Fotemustine | 0.012303 |
| Dexamethasone | Thalidomide | Motexafin gadolinium | 0.012303 |
| Dexamethasone | Thalidomide | Arsenic Trioxide | 0.012303 |
| Dexamethasone | Thalidomide | Carboxyl amino triazole | 0.012303 |
| Gleevec | Cis Retinoic Acid | CCNU | 0.012327 |
| Dexamethasone | Thalidomide | HSPPC-96 Vaccine | 0.012345 |
| Gleevec | CPT11 | Procarbazine | 0.01237 |
| Dexamethasone | Thalidomide | Topotecan | 0.012525 |
| EMD-121974 | Dexamethasone | Gleevec | 0.012549 |
| Dexamethasone | Enzastaurin | Thalidomide | 0.01273 |
| Prednisone | CCNU | CPT11 | 0.012804 |
| Sorafenib | CCNU | CPT11 | 0.012804 |
| CCNU | CPT11 | Arsenic Trioxide | 0.012804 |
| Hydroxurea | CPT11 | Procarbazine | 0.012913 |
| Celebrex | Hydroxurea | CCNU | 0.012945 |
| BCNU | EMD-121974 | CCNU | 0.013031 |
| CCNU | CPT11 | Fotemustine | 0.013117 |
| Tamoxifen | EMD-121974 | Dexamethasone | 0.013271 |
| CCNU | CPT11 | Dendriticcell Vaccine | 0.013302 |
| Cis Retinoic Acid | CCNU | Procarbazine | 0.013307 |
| Gliadel Wafer | Dexamethasone | Antineoplastons | 0.013459 |
| Gleevec | CCNU | Thalidomide | 0.01374 |
| Dexamethasone | Thalidomide | AE788 | 0.013912 |
| Cisplatin | CCNU | Xeloda | 0.013914 |
| Carboplatin | CCNU | Procarbazine | 0.01393 |
| Celebrex | CCNU | Thalidomide | 0.013945 |
| Tamoxifen | BCNU | CCNU | 0.013995 |
| Iressa | Dexamethasone | Thalidomide | 0.014046 |
| Carboplatin | Gleevec | CCNU | 0.01449 |
| Celebrex | Carboplatin | CCNU | 0.014524 |
| BSI-201 | CCNU | CPT11 | 0.014617 |
| Dexamethasone | BSI-201 | Thalidomide | 0.01486 |
| Hydroxurea | CCNU | Antineoplastons | 0.014887 |
| Dexamethasone | Suramin | Thalidomide | 0.015079 |
| Dexamethasone | Thalidomide | MGI-114 | 0.015079 |
| CCNU | CPT11 | Vincristine | 0.015186 |
| Accutane | Hydroxurea | CCNU | 0.015251 |
| Dexamethasone | Thalidomide | Recentin | 0.015308 |
| Suramin | CCNU | CPT11 | 0.015518 |
| CCNU | CPT11 | MGI-114 | 0.015518 |
| Celebrex | Dexamethasone | CI980 | 0.015739 |
| CCNU | Thalidomide | Procarbazine | 0.015849 |
| EMD-121974 | Dexamethasone | Vincristine | 0.015871 |
| CCNU | Thalidomide | Antineoplastons | 0.016007 |
| O6BG | Dexamethasone | Thalidomide | 0.016152 |
| CCNU | CPT11 | AE788 | 0.016338 |
| Celebrex | SAHA | Dexamethasone | 0.016356 |
| 6-Thiguanine | Dexamethasone | Thalidomide | 0.016377 |
| Accutane | CCNU | Thalidomide | 0.016448 |
| Rapamycin | CCNU | CPT11 | 0.016449 |
| Everolimus | CCNU | CPT11 | 0.016449 |
| Chloroquine | CCNU | CPT11 | 0.016449 |
| 6-Mercaptopurine | CCNU | CPT11 | 0.016449 |
| 6-Thiguanine | CCNU | CPT11 | 0.016449 |
| Interferon Alpha | CCNU | CPT11 | 0.016449 |
| O6BG | CCNU | CPT11 | 0.016449 |
| AP23573 | CCNU | CPT11 | 0.016449 |
| Streptozocin | CCNU | CPT11 | 0.016449 |
| CCNU | Topotecan | CPT11 | 0.016449 |
| CCNU | CPT11 | Recentin | 0.016449 |
| CCNU | CPT11 | Motexafin gadolinium | 0.016449 |
| CCNU | CPT11 | Carboxyl amino triazole | 0.016449 |
| Enzastaurin | CCNU | CPT11 | 0.016614 |
| Carboplatin | CCNU | Antineoplastons | 0.016679 |
| Bortezomib | CCNU | CPT11 | 0.016912 |
| CPT11 | Xeloda | Procarbazine | 0.017024 |
| Cisplatin | Gleevec | CCNU | 0.017048 |
| Cisplatin | Celebrex | CCNU | 0.017157 |
| 6-Thiguanine | Dexamethasone | Cyclophosphamide | 0.017159 |
| Dexamethasone | Thalidomide | Dendriticcell Vaccine | 0.017177 |
| Hydroxurea | CCNU | dcVax | 0.017215 |
| Celebrex | Marimastat | Dexamethasone | 0.017409 |
| Sarasar | Hydroxurea | CCNU | 0.017421 |
| Rindopepimut | Dexamethasone | Thalidomide | 0.017464 |
| Rapamycin | CCNU | Procarbazine | 0.017527 |
| Tamoxifen | Dexamethasone | CI980 | 0.017539 |
| Celebrex | IL-13 | Dexamethasone | 0.018151 |
| Tamoxifen | SAHA | Dexamethasone | 0.018195 |
| Celebrex | Daclizumab | Dexamethasone | 0.01825 |
| Celebrex | Dexamethasone | Angiocept | 0.01825 |
| BCNU | CPT11 | Procarbazine | 0.018257 |
| CCNU | CPT11 | HSPPC-96 Vaccine | 0.018439 |
| Celebrex | Dexamethasone | Panzem | 0.018555 |
| CCNU | Thalidomide | dcVax | 0.018597 |
| Sarasar | CCNU | Thalidomide | 0.018694 |
| Gleevec | CPT11 | Vincristine | 0.018744 |
| AMG 102 | CCNU | CPT11 | 0.018902 |
| BCNU | CCNU | Etoposide | 0.019301 |
| Tamoxifen | Marimastat | Dexamethasone | 0.019312 |
| Carboplatin | Sarasar | CCNU | 0.019488 |
| Cisplatin | CCNU | Antineoplastons | 0.01965 |
| Panzem | CCNU | CPT11 | 0.019845 |
| CRA | CCNU | CPT11 | 0.019914 |
| Celebrex | Everolimus | Dexamethasone | 0.019922 |
| Celebrex | AMG 102 | Dexamethasone | 0.020048 |
| Daclizumab | CCNU | CPT11 | 0.02005 |
| CCNU | CPT11 | Angiocept | 0.02005 |
| Tamoxifen | IL-13 | Dexamethasone | 0.020097 |
| IL-13 | CCNU | CPT11 | 0.020118 |
| Cisplatin | Accutane | CCNU | 0.020129 |
| Tamoxifen | Daclizumab | Dexamethasone | 0.020201 |
| Tamoxifen | Dexamethasone | Angiocept | 0.020201 |
| Tamoxifen | CRA | Dexamethasone | 0.020415 |
| Tamoxifen | Dexamethasone | Panzem | 0.020525 |
| Marimastat | CCNU | CPT11 | 0.020642 |
| Carboplatin | Hydroxurea | CCNU | 0.020871 |
| Dexamethasone | Vincristine | CI980 | 0.020944 |
| Cis Retinoic Acid | Hydroxurea | CCNU | 0.021227 |
| SAHA | CCNU | CPT11 | 0.021441 |
| Prednisone | Hydroxurea | CCNU | 0.021638 |
| Sorafenib | Hydroxurea | CCNU | 0.021638 |
| Hydroxurea | CCNU | Arsenic Trioxide | 0.021638 |
| SAHA | Dexamethasone | Vincristine | 0.021725 |
| CCNU | CPT11 | CI980 | 0.021942 |
| Tamoxifen | Everolimus | Dexamethasone | 0.021967 |
| Tamoxifen | AMG 102 | Dexamethasone | 0.022099 |
| Cisplatin | Dexamethasone | Thalidomide | 0.022124 |
| Hydroxurea | CCNU | Dendriticcell Vaccine | 0.022187 |
| CCNU | CPT11 | 81C6 | 0.022248 |
| Celebrex | Dexamethasone | HSPPC-96 Vaccine | 0.022323 |
| Hydroxurea | CCNU | Fotemustine | 0.022399 |
| Cis Retinoic Acid | CCNU | Thalidomide | 0.02252 |
| CCNU | CPT11 | Cyclophosphamide | 0.02258 |
| Cisplatin | CCNU | dcVax | 0.022655 |
| Celebrex | Dexamethasone | Topotecan | 0.0227 |
| Cisplatin | Sarasar | CCNU | 0.022889 |
| Marimastat | Dexamethasone | Vincristine | 0.023054 |
| Hydroxurea | CCNU | Thalidomide | 0.023201 |
| Gliadel Wafer | Dexamethasone | Avastin | 0.023205 |
| Prednisone | CCNU | Thalidomide | 0.023221 |
| Sorafenib | CCNU | Thalidomide | 0.023221 |
| CCNU | Thalidomide | Arsenic Trioxide | 0.023221 |
| Hydroxurea | CPT11 | Vincristine | 0.023248 |
| Rindopepimut | Hydroxurea | CCNU | 0.023724 |
| Carboplatin | Cis Retinoic Acid | CCNU | 0.023752 |
| Dexamethasone | Gleevec | HSPPC-96 Vaccine | 0.023775 |
| EMD-121974 | CCNU | CPT11 | 0.02384 |
| CCNU | Thalidomide | Dendriticcell Vaccine | 0.023876 |
| Cisplatin | CCNU | Procarbazine | 0.023905 |
| Dexamethasone | Cis Retinoic Acid | Thalidomide | 0.023908 |
| CCNU | Thalidomide | Fotemustine | 0.023954 |
| IL-13 | Dexamethasone | Vincristine | 0.023988 |
| Daclizumab | Dexamethasone | Vincristine | 0.024112 |
| Dexamethasone | Vincristine | Angiocept | 0.024112 |
| Gliadel Wafer | Dexamethasone | Thalidomide | 0.024275 |
| BSI-201 | Hydroxurea | CCNU | 0.024297 |
| Rapamycin | Gliadel Wafer | Dexamethasone | 0.024337 |
| CRA | Dexamethasone | Vincristine | 0.024366 |
| Tamoxifen | Dexamethasone | HSPPC-96 Vaccine | 0.024388 |
| Dexamethasone | Panzem | Vincristine | 0.024497 |
| Oxaliplatin | Tamoxifen | Dexamethasone | 0.024672 |
| Tamoxifen | Rapamcyin | Dexamethasone | 0.024672 |
| Tamoxifen | 6-Mercaptopurine | Dexamethasone | 0.024672 |
| Tamoxifen | Dexamethasone | Prednisone | 0.024672 |
| Tamoxifen | Dexamethasone | Suramin | 0.024672 |
| Tamoxifen | Dexamethasone | Bortezomib | 0.024672 |
| Tamoxifen | Dexamethasone | Streptozocin | 0.024672 |
| Tamoxifen | Dexamethasone | CPT11 | 0.024672 |
| Tamoxifen | Dexamethasone | Fotemustine | 0.024672 |
| Tamoxifen | Dexamethasone | MGI-114 | 0.024672 |
| Tamoxifen | Dexamethasone | Motexafin gadolinium | 0.024672 |
| Tamoxifen | Dexamethasone | Arsenic Trioxide | 0.024672 |
| Tamoxifen | Dexamethasone | Carboxyl amino triazole | 0.024672 |
| Carboplatin | CCNU | Dendriticcell Vaccine | 0.024725 |
| Tamoxifen | Dexamethasone | Topotecan | 0.024887 |
| Gliadel Wafer | Erlotinib | Dexamethasone | 0.024894 |
| O6BG | Hydroxurea | CCNU | 0.024931 |
| Carboplatin | CCNU | Fotemustine | 0.024995 |
| Celebrex | Dexamethasone | AE788 | 0.025061 |
| Dexamethasone | Thalidomide | dcVax | 0.025106 |
| Tamoxifen | Dexamethasone | Enzastaurin | 0.025253 |
| Celebrex | Iressa | Dexamethasone | 0.025288 |
| Rindopepimut | CCNU | Thalidomide | 0.025425 |
| CPT11 | Vincristine | Xeloda | 0.025481 |
| Hydroxurea | CCNU | Vincristine | 0.02549 |
| CPT11 | Etoposide | Procarbazine | 0.025582 |
| Carboplatin | 6-Thiguanine | Dexamethasone | 0.025655 |
| 6-Mercaptopurine | Hydroxurea | CCNU | 0.025672 |
| Hydroxurea | Streptozocin | CCNU | 0.025672 |
| CCNU | Thalidomide | Cyclophosphamide | 0.025866 |
| Gliadel Wafer | Dexamethasone | Hydroxurea | 0.025959 |
| Suramin | Hydroxurea | CCNU | 0.026025 |
| Hydroxurea | CCNU | MGI-114 | 0.026025 |
| Dexamethasone | Cis Retinoic Acid | Cyclophosphamide | 0.026089 |
| BSI-201 | CCNU | Thalidomide | 0.02611 |
| Gliadel Wafer | Dexamethasone | Xeloda | 0.026197 |
| Everolimus | Dexamethasone | Vincristine | 0.026211 |
| AMG 102 | Dexamethasone | Vincristine | 0.026368 |
| Oxaliplatin | Hydroxurea | CCNU | 0.026448 |
| Carboplatin | Gliadel Wafer | Dexamethasone | 0.02658 |
| Rapamycin | Cis Retinoic Acid | CCNU | 0.026581 |
| O6BG | CCNU | Thalidomide | 0.0267 |
| Gliadel Wafer | Dexamethasone | Cyclophosphamide | 0.026745 |
| Celebrex | Dexamethasone | BSI-201 | 0.026751 |
| Celebrex | Dexamethasone | Enzastaurin | 0.02689 |
| EMD-121974 | Dexamethasone | Procarbazine | 0.027029 |
| Celebrex | Dexamethasone | Suramin | 0.027036 |
| Celebrex | Dexamethasone | MGI-114 | 0.027036 |
| Iressa | Hydroxurea | CCNU | 0.027186 |
| CCNU | Thalidomide | Vincristine | 0.027289 |
| Hydroxurea | CCNU | AE788 | 0.027345 |
| Tamoxifen | Dexamethasone | AE788 | 0.027358 |
| CCNU | CPT11 | Avastin | 0.027371 |
| Rapamycin | EMD-121974 | Dexamethasone | 0.027451 |
| Celebrex | Dexamethasone | Recentin | 0.02751 |
| Iressa | Tamoxifen | Dexamethasone | 0.027595 |
| Cisplatin | Cis Retinoic Acid | CCNU | 0.027678 |
| Cis Retinoic Acid | CPT11 | Procarbazine | 0.027714 |
| Suramin | CCNU | Thalidomide | 0.027853 |
| CCNU | Thalidomide | MGI-114 | 0.027853 |
| Enzastaurin | Hydroxurea | CCNU | 0.028085 |
| Oxaliplatin | CCNU | Thalidomide | 0.028217 |
| Cisplatin | Prednisone | CCNU | 0.028263 |
| Cisplatin | Sorafenib | CCNU | 0.028263 |
| Cisplatin | CCNU | Arsenic Trioxide | 0.028263 |
| Bortezomib | Hydroxurea | CCNU | 0.028266 |
| BCNU | CCNU | Avastin | 0.028362 |
| Cisplatin | Hydroxurea | CCNU | 0.028625 |
| Dexamethasone | Gleevec | Enzastaurin | 0.028651 |
| Carboplatin | CCNU | Thalidomide | 0.028662 |
| Tamoxifen | Gleevec | CCNU | 0.028768 |
| Celebrex | O6BG | Dexamethasone | 0.028841 |
| Cisplatin | CCNU | Dendriticcell Vaccine | 0.028979 |
| CCNU | Thalidomide | CPT11 | 0.029024 |
| Iressa | CCNU | Thalidomide | 0.029076 |
| Dexamethasone | Vincristine | HSPPC-96 Vaccine | 0.0291 |
| Hydroxurea | CCNU | Topotecan | 0.029125 |
| Rapamcyin | CCNU | Thalidomide | 0.029205 |
| Chloroquine | CCNU | Thalidomide | 0.029205 |
| 6-Mercaptopurine | CCNU | Thalidomide | 0.029205 |
| 6-Thiguanine | CCNU | Thalidomide | 0.029205 |
| Interferon Alpha | CCNU | Thalidomide | 0.029205 |
| AP23573 | CCNU | Thalidomide | 0.029205 |
| Streptozocin | CCNU | Thalidomide | 0.029205 |
| CCNU | Thalidomide | Recentin | 0.029205 |
| CCNU | Thalidomide | Motexafin gadolinium | 0.029205 |
| CCNU | Thalidomide | Carboxyl amino triazole | 0.029205 |
| BCNU | Gleevec | CPT11 | 0.02922 |
| Tamoxifen | Dexamethasone | BSI-201 | 0.029242 |
| CCNU | Thalidomide | AE788 | 0.029244 |
| EMD-121974 | Erlotinib | Dexamethasone | 0.029295 |
| Rapamycin | CCNU | Thalidomide | 0.029458 |
| Carboplatin | Oxaliplatin | CCNU | 0.029463 |
| Celebrex | Gliadel Wafer | Dexamethasone | 0.029523 |
| EMD-121974 | Dexamethasone | Hydroxurea | 0.029675 |
| Dexamethasone | Topotecan | Vincristine | 0.029677 |
| Tamoxifen | Dexamethasone | Recentin | 0.030035 |
| Bortezomib | CCNU | Thalidomide | 0.030214 |
| AP23573 | Dexamethasone | Thalidomide | 0.030351 |
| BCNU | Hydroxurea | CPT11 | 0.030381 |
| Sarasar | Dexamethasone | Thalidomide | 0.030431 |
| Celebrex | Dexamethasone | Dendriticcell Vaccine | 0.030656 |
| Hydroxurea | CCNU | HSPPC-96 Vaccine | 0.030713 |
| BCNU | Erlotinib | CCNU | 0.030761 |
| 6-Thiguanine | Dexamethasone | Gleevec | 0.030823 |
| Enzastaurin | CCNU | Thalidomide | 0.030824 |
| Tamoxifen | CCNU | Procarbazine | 0.030845 |
| Tamoxifen | 6-Thiguanine | Dexamethasone | 0.030891 |
| Cisplatin | Rindopepimut | CCNU | 0.0309 |
| Celebrex | Rindopepimut | Dexamethasone | 0.031037 |
| CCNU | Thalidomide | Topotecan | 0.031117 |
| Rapamycin | Hydroxurea | CCNU | 0.031238 |
| Tamoxifen | Gliadel Wafer | Dexamethasone | 0.031269 |
| Tamoxifen | O6BG | Dexamethasone | 0.031298 |
| AMG 102 | Hydroxurea | CCNU | 0.031455 |
| Gliadel Wafer | Dexamethasone | Gleevec | 0.031555 |
| Cisplatin | BSI-201 | CCNU | 0.031647 |
| Cisplatin | CCNU | Thalidomide | 0.032116 |
| Cisplatin | O6BG | CCNU | 0.032424 |
| Dexamethasone | Vincristine | AE788 | 0.032606 |
| Dexamethasone | Gleevec | Dendriticcell Vaccine | 0.032633 |
| CCNU | Thalidomide | HSPPC-96 Vaccine | 0.032788 |
| Sarasar | Dexamethasone | Xeloda | 0.032789 |
| Iressa | Dexamethasone | Vincristine | 0.032887 |
| Hydroxurea | Panzem | CCNU | 0.032959 |
| CRA | Hydroxurea | CCNU | 0.03307 |
| Carboplatin | Sarasar | Dexamethasone | 0.033281 |
| Daclizumab | Hydroxurea | CCNU | 0.033288 |
| Hydroxurea | CCNU | Angiocept | 0.033288 |
| Tamoxifen | Dexamethasone | Dendriticcell Vaccine | 0.033312 |
| CCNU | CPT11 | Etoposide | 0.033377 |
| IL-13 | Hydroxurea | CCNU | 0.033395 |
| Carboplatin | Dexamethasone | Vincristine | 0.03344 |
| AMG 102 | CCNU | Thalidomide | 0.033567 |
| Rindopepimut | Tamoxifen | Dexamethasone | 0.033577 |
| Everolimus | CCNU | Thalidomide | 0.033694 |
| Cisplatin | Suramin | CCNU | 0.033801 |
| Cisplatin | CCNU | MGI-114 | 0.033801 |
| Cisplatin | Carboplatin | CCNU | 0.03399 |
| Marimastat | Hydroxurea | CCNU | 0.034231 |
| BCNU | CPT11 | Vincristine | 0.034429 |
| Chloroquine | Dexamethasone | Thalidomide | 0.034564 |
| Dexamethasone | Enzastaurin | Vincristine | 0.034744 |
| Dexamethasone | BSI-201 | Vincristine | 0.034821 |
| Dexamethasone | CI980 | Procarbazine | 0.034955 |
| Dexamethasone | Suramin | Vincristine | 0.035045 |
| Dexamethasone | Vincristine | MGI-114 | 0.035045 |
| Panzem | CCNU | Thalidomide | 0.035147 |
| Cisplatin | Iressa | CCNU | 0.035261 |
| CRA | CCNU | Thalidomide | 0.035263 |
| Cisplatin | CCNU | AE788 | 0.035461 |
| Daclizumab | CCNU | Thalidomide | 0.035492 |
| CCNU | Thalidomide | Angiocept | 0.035492 |
| SAHA | Hydroxurea | CCNU | 0.035501 |
| EMD-121974 | Dexamethasone | Etoposide | 0.035596 |
| IL-13 | CCNU | Thalidomide | 0.035605 |
| Dexamethasone | Vincristine | Recentin | 0.035759 |
| Erlotinib | Dexamethasone | Cyclophosphamide | 0.035955 |
| SAHA | Dexamethasone | Procarbazine | 0.036121 |
| Hydroxurea | CCNU | CI980 | 0.036299 |
| Cisplatin | Enzastaurin | CCNU | 0.036366 |
| Marimastat | CCNU | Thalidomide | 0.036481 |
| CCNU | Etoposide | Xeloda | 0.036518 |
| Cisplatin | Oxaliplatin | CCNU | 0.036618 |
| Cisplatin | Bortezomib | CCNU | 0.036618 |
| Dexamethasone | Cis Retinoic Acid | Xeloda | 0.03672 |
| 6-Thiguanine | Dexamethasone | Vincristine | 0.036836 |
| Carboplatin | CCNU | Cyclophosphamide | 0.036881 |
| Dexamethasone | Gleevec | Hydroxurea | 0.037173 |
| Carboplatin | Dexamethasone | Cis Retinoic Acid | 0.037237 |
| O6BG | Dexamethasone | Vincristine | 0.037268 |
| Hydroxurea | CCNU | Cyclophosphamide | 0.037311 |
| Celebrex | Sarasar | Dexamethasone | 0.037311 |
| Gliadel Wafer | Dexamethasone | Vincristine | 0.037312 |
| Erlotinib | Dexamethasone | CI980 | 0.037319 |
| Dexamethasone | Thalidomide | Antineoplastons | 0.037402 |
| Cisplatin | CCNU | Topotecan | 0.037694 |
| SAHA | CCNU | Thalidomide | 0.037814 |
| EMD-121974 | Dexamethasone | Avastin | 0.037872 |
| Marimastat | Dexamethasone | Procarbazine | 0.038094 |
| Dexamethasone | Hydroxurea | CI980 | 0.03825 |
| SAHA | Erlotinib | Dexamethasone | 0.038447 |
| CCNU | Thalidomide | CI980 | 0.038649 |
| Cisplatin | Celebrex | Dexamethasone | 0.038746 |
| BCNU | CPT11 | Xeloda | 0.039027 |
| Hydroxurea | CCNU | 81C6 | 0.039179 |
| Dexamethasone | Gleevec | Cyclophosphamide | 0.039205 |
| Dexamethasone | Thalidomide | Cyclophosphamide | 0.039408 |
| IL-13 | Dexamethasone | Procarbazine | 0.039474 |
| SAHA | Dexamethasone | Hydroxurea | 0.039503 |
| Dexamethasone | Vincristine | Dendriticcell Vaccine | 0.039634 |
| Daclizumab | Dexamethasone | Procarbazine | 0.039657 |
| Dexamethasone | Angiocept | Procarbazine | 0.039657 |
| Cisplatin | CCNU | HSPPC-96 Vaccine | 0.039684 |
| Tamoxifen | Sarasar | Dexamethasone | 0.039795 |
| Rindopepimut | Dexamethasone | Vincristine | 0.039961 |
| CRA | Dexamethasone | Procarbazine | 0.040032 |
| Dexamethasone | Panzem | Procarbazine | 0.040223 |
| Tamoxifen | CCNU | Thalidomide | 0.040285 |
| Dexamethasone | Avastin | Cyclophosphamide | 0.040285 |
| Celebrex | Interferon Alpha | Dexamethasone | 0.040325 |
| Celebrex | Dexamethasone | Sorafenib | 0.040325 |
| Marimastat | Erlotinib | Dexamethasone | 0.040349 |
| CPT11 | Etoposide | Vincristine | 0.040432 |
| Cisplatin | AMG 102 | CCNU | 0.040612 |
| Cisplatin | Everolimus | CCNU | 0.040762 |
| Cisplatin | Tamoxifen | Dexamethasone | 0.041539 |
| CCNU | Thalidomide | 81C6 | 0.041557 |
| Dexamethasone | CI980 | Avastin | 0.0416 |
| Marimastat | Dexamethasone | Hydroxurea | 0.041621 |
| IL-13 | Erlotinib | Dexamethasone | 0.041676 |
| Daclizumab | Erlotinib | Dexamethasone | 0.041852 |
| Erlotinib | Dexamethasone | Angiocept | 0.041852 |
| CRA | Erlotinib | Dexamethasone | 0.042211 |
| 6-Thiguanine | CPT11 | Procarbazine | 0.04233 |
| Erlotinib | Dexamethasone | Panzem | 0.042395 |
| Cisplatin | Panzem | CCNU | 0.04249 |
| Cisplatin | CRA | CCNU | 0.042628 |
| SAHA | Dexamethasone | Avastin | 0.042683 |
| Gleevec | CCNU | Etoposide | 0.042702 |
| Everolimus | Dexamethasone | Procarbazine | 0.042737 |
| Cisplatin | CCNU | Cyclophosphamide | 0.042742 |
| Cisplatin | Daclizumab | CCNU | 0.0429 |
| Cisplatin | CCNU | Angiocept | 0.0429 |
| AMG 102 | Dexamethasone | Procarbazine | 0.042966 |
| Cisplatin | IL-13 | CCNU | 0.043035 |
| IL-13 | Dexamethasone | Hydroxurea | 0.043102 |
| Tamoxifen | Interferon Alpha | Dexamethasone | 0.043164 |
| Tamoxifen | Dexamethasone | Sorafenib | 0.043164 |
| Tamoxifen | Dexamethasone | Cis Retinoic Acid | 0.043266 |
| Daclizumab | Dexamethasone | Hydroxurea | 0.043298 |
| Dexamethasone | Hydroxurea | Angiocept | 0.043298 |
| Carboplatin | CCNU | 81C6 | 0.043467 |
| Celebrex | CCNU | Etoposide | 0.043681 |
| CRA | Dexamethasone | Hydroxurea | 0.043699 |
| Celebrex | Dexamethasone | dcVax | 0.043752 |
| Dexamethasone | Gleevec | Cis Retinoic Acid | 0.04384 |
| Dexamethasone | Hydroxurea | Panzem | 0.043905 |
| Cisplatin | Marimastat | CCNU | 0.044076 |
| Marimastat | Dexamethasone | Avastin | 0.044504 |
| Dexamethasone | Etoposide | CI980 | 0.044741 |
| Everolimus | Erlotinib | Dexamethasone | 0.0448 |
| AMG 102 | Erlotinib | Dexamethasone | 0.045019 |
| Tamoxifen | Cis Retinoic Acid | CCNU | 0.045458 |
| Cisplatin | SAHA | CCNU | 0.045658 |
| IL-13 | Dexamethasone | Avastin | 0.045769 |
| EMD-121974 | Hydroxurea | CCNU | 0.04583 |
| SAHA | Dexamethasone | Etoposide | 0.045981 |
| CRA | Dexamethasone | Avastin | 0.046277 |
| 6-Thiguanine | Dexamethasone | 81C6 | 0.04638 |
| Dexamethasone | Panzem | Avastin | 0.046452 |
| Dexamethasone | Procarbazine | HSPPC-96 Vaccine | 0.046595 |
| Cisplatin | CCNU | CI980 | 0.04665 |
| Tamoxifen | Dexamethasone | dcVax | 0.046838 |
| AMG 102 | Dexamethasone | Hydroxurea | 0.046843 |
| Gliadel Wafer | Dexamethasone | 81C6 | 0.046898 |
| Tamoxifen | Hydroxurea | CCNU | 0.046968 |
| Sarasar | Dexamethasone | Vincristine | 0.047311 |
| Oxaliplatin | Dexamethasone | Procarbazine | 0.047738 |
| Rapamcyin | Dexamethasone | Procarbazine | 0.047738 |
| 6-Mercaptopurine | Dexamethasone | Procarbazine | 0.047738 |
| Dexamethasone | Prednisone | Procarbazine | 0.047738 |
| Dexamethasone | Bortezomib | Procarbazine | 0.047738 |
| Dexamethasone | Streptozocin | Procarbazine | 0.047738 |
| Dexamethasone | Fotemustine | Procarbazine | 0.047738 |
| Dexamethasone | Procarbazine | Motexafin gadolinium | 0.047738 |
| Dexamethasone | Procarbazine | Arsenic Trioxide | 0.047738 |
| Dexamethasone | Procarbazine | Carboxyl amino triazole | 0.047738 |
| Dexamethasone | Topotecan | Procarbazine | 0.047769 |
| Rapamycin | Dexamethasone | HSPPC-96 Vaccine | 0.047914 |
| CCNU | Etoposide | Procarbazine | 0.048486 |
| EMD-121974 | CCNU | Thalidomide | 0.048494 |
| Celebrex | Dexamethasone | Cis Retinoic Acid | 0.048742 |
| AMG 102 | Dexamethasone | Avastin | 0.048936 |
| CCNU | Etoposide | Antineoplastons | 0.049237 |
| Cisplatin | Dexamethasone | Vincristine | 0.049345 |
| Carboplatin | Erlotinib | Dexamethasone | 0.049345 |
| IL-13 | Dexamethasone | Etoposide | 0.049516 |
| Erlotinib | Dexamethasone | Topotecan | 0.049588 |
| Oxaliplatin | Erlotinib | Dexamethasone | 0.049942 |
| Rapamcyin | Erlotinib | Dexamethasone | 0.049942 |
| Rapamycin | Erlotinib | Dexamethasone | 0.049942 |
| Chloroquine | Erlotinib | Dexamethasone | 0.049942 |
| 6-Mercaptopurine | Erlotinib | Dexamethasone | 0.049942 |
| Interferon Alpha | Erlotinib | Dexamethasone | 0.049942 |
| AP23573 | Erlotinib | Dexamethasone | 0.049942 |
| Erlotinib | Dexamethasone | Prednisone | 0.049942 |
| Erlotinib | Dexamethasone | Bortezomib | 0.049942 |
| Erlotinib | Dexamethasone | Sorafenib | 0.049942 |
| Erlotinib | Dexamethasone | Streptozocin | 0.049942 |
| Erlotinib | Dexamethasone | Fotemustine | 0.049942 |
| Erlotinib | Dexamethasone | HSPPC-96 Vaccine | 0.049942 |
| Erlotinib | Dexamethasone | Motexafin gadolinium | 0.049942 |
| Erlotinib | Dexamethasone | Arsenic Trioxide | 0.049942 |
| Erlotinib | Dexamethasone | Carboxyl amino triazole | 0.049942 |
