# Supplementary material for: Gene expression and network-based analysis reveals a novel role for hsa-miR-9 and drug control over the p38 network in glioblastoma multiforme progression
Source: Genome Med. 2011 Nov 28;3(11):77. doi: 10.1186/gm293 (PMC3308032; doi:10.1186/gm293)
Supplement: Additional file 2 — Table S2 - all drugs with their corresponding gene targets. This table also indicates whether there is a connection or not between a drug and the p38 pathway, and provides the number of patients who received each drug. [file gm293-S2.PPT]

## Slide 1
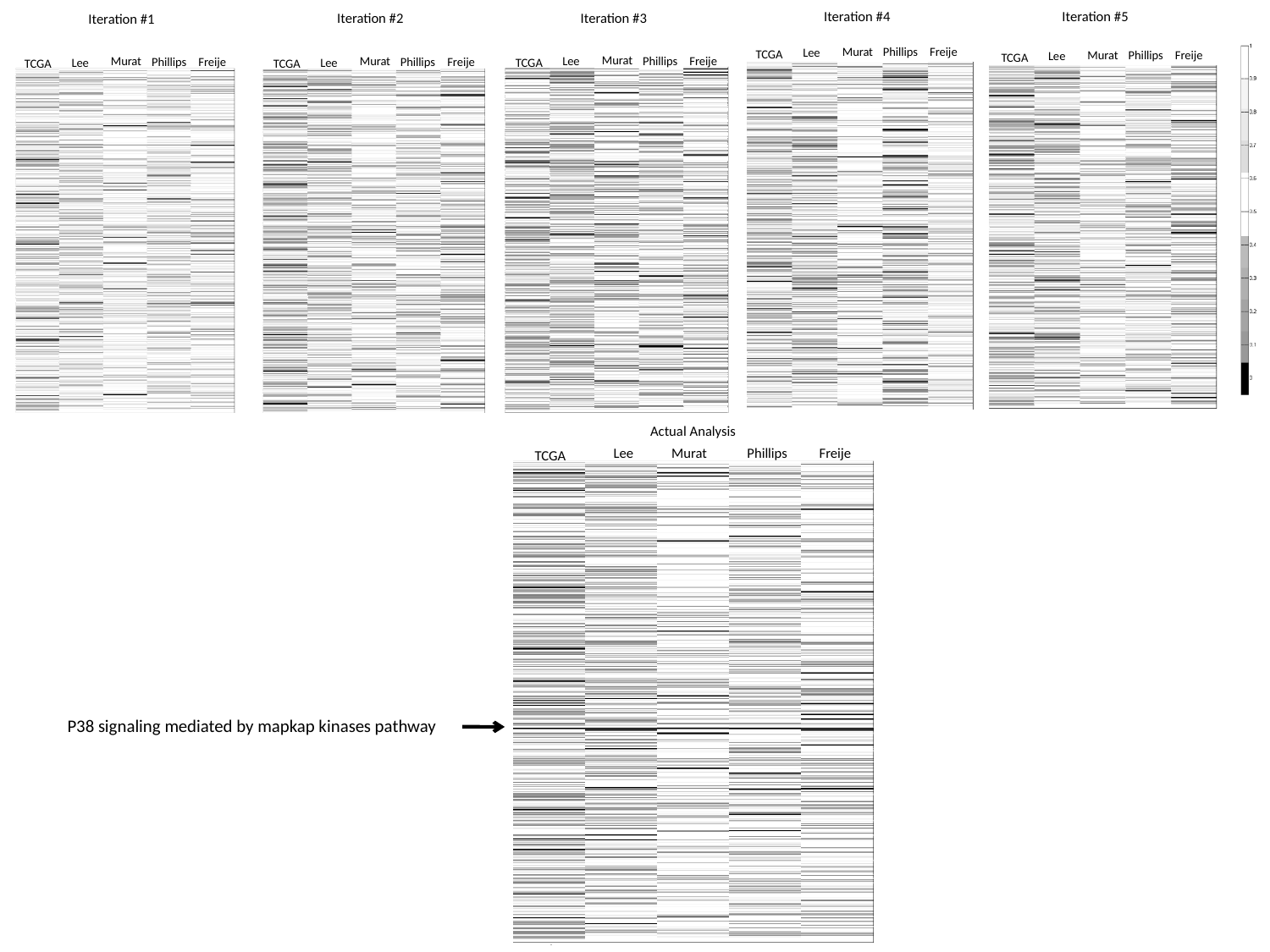

Iteration #4
Iteration #5
Iteration #2
Iteration #3
Iteration #1
TCGA
Murat
Phillips
Freije
Lee
TCGA
Murat
Phillips
Freije
Lee
TCGA
Murat
Phillips
Freije
Lee
TCGA
Murat
Phillips
Freije
Lee
TCGA
Murat
Phillips
Freije
Lee
TCGA
Lee
Murat
Phillips
Freije
P38 signaling mediated by mapkap kinases pathway
Actual Analysis
