# Supplementary material for: Gene expression and network-based analysis reveals a novel role for hsa-miR-9 and drug control over the p38 network in glioblastoma multiforme progression
Source: Genome Med. 2011 Nov 28;3(11):77. doi: 10.1186/gm293 (PMC3308032; doi:10.1186/gm293)
Supplement: Additional file 3 — Figure S1 - heat maps describing the false discovery rate analysis. The five heat maps illustrate the five iterations that were performed. Every row represents a different pathway (overall 579 pathways), and columns represent the five datasets tested. A black line indicates a significant P-value (< 0.05) in Kaplan-Meier survival analysis. The heat map at the bottom shows the actual analysis that was performed with the p38 pathway as significant for survival in all five sets. [file gm293-S3.DOC]

| Drug Name | Target | P38 targets | Number of Patients received the drug |
| --- | --- | --- | --- |
| Temozolomide | MGMT | No | 184 |
| Avastin | VEGFR | No | 45 |
| CCNU | STMN4 | Yes | 38 |
| Dexamethasone | NR3C1 | No | 37 |
| CPT11 | TOP1MT | No | 31 |
| Etoposide | TOP2A | No | 23 |
| Gliadel Wafer | GSR | No | 20 |
| Erlotinib | NR1I2 , EGFR | No | 18 |
| BCNU | GSR | No | 17 |
| Procarbazine | XDH | No | 13 |
| Thalidomide | TNF | No | 12 |
| Hydroxurea | RRM1 | No | 10 |
| Tamoxifen | ESR1 | Yes | 10 |
| Vincristine | TUBB | No | 9 |
| Carboplatin | DNA | No | 8 |
| Celebrex | COX2 | Yes | 8 |
| Gleevec | PDGFRA | No | 8 |
| Cisplatin | ABCC3 | No | 7 |
| IL-13 | IL13 | No | 7 |
| Rapamcyin | mTOR | No | 7 |
| 81C6 | - | - | 6 |
| Cis Retinoic Acid | RARA | Yes | 6 |
| Xeloda | TYMS | NO | 6 |
| 6-Thiguanine | HPRT1 | No | 5 |
| Cyclophosphamide | - | No | 4 |
| Sarasar | FNTA | No | 3 |
| Accutane | RARA | YES | 2 |
| Antineoplastons | - | - | 2 |
| Dendriticcell Vaccine | - | No | 2 |
| EMD-121974 | ITGAV | - | 2 |
| Enzastaurin | PKC | No | 2 |
| Fotemustine | TXNRD1 | No | 2 |
| HSPPC-96 Vaccine | - | No | 2 |
| Oxaliplatin | DNA synthesis | No | 2 |
| Sorafenib | RAF1 | Yes | 2 |
| 6-Mercaptopurine | HPRT1 | No | 1 |
| AE788 | VEGFR | No | 1 |
| AMG 102 | HGF | No | 1 |
| Angiocept | VEGFR | No | 1 |
| AP23573 | mTOR | No | 1 |
| Arsenic Trioxide | - | - | 1 |
| Bortezomib | PSMD2 | No | 1 |
| BSI-201 | PARP1 | NO | 1 |
| Carboxyl amino triazole | - | - | 1 |
| Chloroquine | GSTA2 | No | 1 |
| CI980 | TUBB | No | 1 |
| CRA | HDAC | No | 1 |
| Daclizumab | IL2RA | No | 1 |
| dcVax | - | - | 1 |
| Everolimus | mTOR | No | 1 |
| Interferon Alpha | IFNA1 | No | 1 |
| Iressa | EGFR | No | 1 |
| Marimastat | MMP1 | No | 1 |
| MGI-114 | - | - | 1 |
| Motexafin gadolinium | TrxR | No | 1 |
| O6BG | MGMT | No | 1 |
| Panzem | SOD | No | 1 |
| Prednisone | NR3C1 | No | 1 |
| Recentin | VEGFR | No | 1 |
| Rindopepimut | EGFR | No | 1 |
| SAHA | HDAC1 | No | 1 |
| Streptozocin | SLC2A2 | No | 1 |
| Suramin | P2RY2 | No | 1 |
| Topotecan | TOP1 | No | 1 |
